# Implementing a connected health intervention for remote patient monitoring in Saudi Arabia and Pakistan: explaining ‘the what’ and ‘the how’

**DOI:** 10.1186/s12992-019-0462-1

**Published:** 2019-03-08

**Authors:** Abdullah M. Aldahmash, Zakiuddin Ahmed, Fatima R. Qadri, Subash Thapa, Abdulrahman Mohammed AlMuammar

**Affiliations:** 1 Prince Naif Bin Abdulaziz Health Research Center, King Saud University, Riyadh, Saudi Arabia; 2 Research Unit of General Practice, Department of Public Health, University of Southern Denmark, Odense, Denmark; 3 King Saud University Medical City, Riyadh, Saudi Arabia

**Keywords:** Connected health, Implementation science, Remote patient monitoring, Saudi Arabia, Pakistan

## Abstract

**Background:**

Recent developments in connected health technology provide an opportunity to remotely monitor and provide health care to the patient needing long-term medical care. However, information about how any connected health interventions should be implemented for remote patient monitoring, and how patients should be educated and enabled for active participation in treatment is still not available to a sufficient degree.

**Discussion:**

In this paper, we discussed what the components of a connected health intervention, entitled Remotely Accessible Health Care at Home (RAHAH), are, and how this intervention has been implemented in Saudi Arabia and Pakistan. The aim of this intervention is to remotely monitor, treat and educate patients needing long-term medical care. The description of the intervention is presented based on the Template for Intervention Description and Replication (TIDieR) checklist for the transparent, comprehensive and explicit reporting.

**Conclusion:**

We believe, successful implementation of RAHAH would be crucial to monitor and manage growing chronic care populations more effectively and efficiently in Saudi Arabia and Pakistan.

## Introduction

Most people with chronic diseases (e.g., diabetes, heart diseases) or certain vulnerable groups (e.g., the elderly and women with high-risk pregnancies) would require long-term medical care, which involves a variety of medical services (e.g., continuous health monitoring, treatment, moral support and health education) designed to meet a person’s health or personal care needs over a long-term period. In many countries, health care is still practiced conventionally, and the patients needing long-standing medical care are routinely examined in a clinical setting. [1]. It is noted that providing chronic care based on a conventional clinic set up to population sub-groups would incur more costs to healthcare managers [2]. It is generally because long-term medical care would increase the rate of hospitalizations, which, in turn, would increase the demand of hospital beds, regular consultations, medical procedures and hospital meals. Additionally, extended hospitalization has been associated with numerous complications for the patient such as hospital-acquired infections, morbidity, mortality [3], and adverse drug events [4].

Remote patient monitoring, on the other hand, can save some costs associated with long term medical care by enabling healthcare providers to monitor patients outside conventional clinical settings using technology and digital devices, especially for those patients with chronic conditions and in need long-term medical care [5]. With recent advances in the use of internet, smart mobile phones and computers, and along with increased digital literacy globally, the prospect for patient monitoring outside traditional clinical set up by using digital devices has become a possibility [6]. This process is conceptualized as ‘connected health’, since it provides a model for health management where patients and physicians are connected by means of timely monitoring, sharing and presentation of accurate and pertinent information regarding patient status through smarter use of data, technology and communication platforms [7, 8].

Several interventions have tested and made use of digital heath devices and some of the examples include, for instance, a mobile phone-based, home-based exercise program [9], mobile phone-based retention in HIV care [10] and internet-based mental health interventions [11]. These interventions have highlighted the success of digital health technologies (e.g., internet, mobile phones and tablets) in remotely monitoring the patients and increasing their adherence to the treatment [9–11]. While these interventions have made valuable contributions to an evolving field, that is connected health, it may also be argued that these interventions lacked a coordinated and effective use of all the digital health devices and technologies which are available these days.

In other words, program managers have not been able to make the optimal use of all the emerging digital devices in an organized and systematic way, which could in fact be proven very efficient and effective to manage the growing chronic care patients. Additionally, there lacks evidence suggesting how these various digital health devices can be integrated and connected into clinical care to develop absolute connected health interventions, which can be used to monitor, treat and educate the patients for their active participation in the treatment [12]. In this paper, we intend to describe and explain the components of a connected health intervention, and illustrate how it is being implemented in a multi-country setting.

## Main text

In this paper, we provided the description of a connected health intervention, named Remotely Accessible Healthcare at Home (RAHAH) using some of the applicable items from Template for Intervention Description and Replication (TIDieR) checklist. TIDieR checklist provides a standardized template for authors to describe some of the very important elements for reporting of a health-related intervention, and this checklist was developed by an international team of experts to promote full and accurate description of interventions [13]. RAHAH is aimed at remotely monitoring, treating and educating the patients needing long-term care through the use of connected health tools. RAHAH was first developed in April 2017 and it is in an initial stage of implementation. It is a multi-country intervention that is currently being implemented in Saudi Arabia and Pakistan under the support and leadership of Prince Naif Bin AbdulAziz Health Research Center, King Saud University, Riyadh, Saudi Arabia.

## Rationale of the elements essential to the intervention

High-income countries have been making some remarkable progress in health care over the years; however, most of them are facing a high burden of non-communicable diseases, and a few including Saudi Arabia, are still lacking effective strategies to provide quality healthcare to manage the growing chronic care population [14]. For instance, several studies from Saudi Arabia have noted that some people who need long term medical care (e.g., diabetes, cancer and heart diseases) may have compromised access to quality health care due to long waiting lists, high burden of hospital acquired infections due to long-term hospitalizations and high rate of medical errors at the hospitals [15–17].

Through the National Transformation Program 2020, the Saudi Arabian government is attempting to effect radical changes in the structure and function of its health care system to achieve quality care and effective service delivery [18]. One of the strategies to fight against the increased burden of the diseases and subsequently improve the health of the people is to institutionalize telemedicine and digital health approaches in Saudi Arabian health care [18]. A routine use of telemedicine has already been implemented in a few specialized hospitals in Saudi Arabia. One of the pioneer examples include the Health Outreach Services Program initiated by King Faisal Specialist Hospital and Research Centre in Riyadh [19]. However, this program did not provide close and robust monitoring of the patients needing long-term care and thus, connected health interventions have not yet been integrated in the routine health care in Saudi Arabia.

Likewise, low- and middle-income countries are also increasingly facing the burden of non-communicable diseases [20], and particularly, South Asian countries, including Pakistan, have been through rapid epidemiological transition facing the double burden of non-communicable (e.g., cardiovascular diseases and diabetes mellitus) and communicable diseases (e.g., HIV, TB and malaria) [21]. Moreover, the access to quality health care is remarkably poor and the patients who need long term care are affected the most [22]. In Pakistan, nearly 64% of the population reside in rural areas that lack specialized healthcare facilities and healthcare facilities in the public sector are facing a shortage of healthcare providers [23].

Pakistan’s National Health Vision 2016–2025 has recognized the key gaps in the health information system and envisioned to incorporate innovative health technologies, such as telemedicine and digital health, for the improvement of overall health care [24]. The Pakistani government has also felt the need to increase investments and developments in the field of information and communication technology, and introduction of innovative connected health interventions in order to increase health care access to those who need it the most [25].

Lately, in both Saudi Arabia and Pakistan, there has been some noteworthy progress in information and communication technology, and the use of internet. Thus, RAHAH has been developed and it is being implemented as a comprehensive connected health intervention in these two different country settings. Given that there are some contextual similarities between these two countries in terms of religion and culture, we believe, RAHAH can be taken as an opportunity to simultaneously apply, compare and learn about the implementation experiences and potential socio-economic contextual differences that could influence the intervention implementation in these two countries. The second author ZA, who is originally from Pakistan and has several years of experience in digital health, has played a collaborative role in implementing RAHAH jointly in these two countries. An intervention that is being implemented simultaneously in a high-income country setting and a low- and middle-income country setting via collaboration and partnership can also be important to create a positive impact on the sharing of knowledge and experience and building the capacity to collectively deal with the implementation challenges [26].

## Intervention location

RAHAH has been robustly implemented in home healthcare and bariatric surgery departments at King Saud University Medical City Hospital (KSUMC), Riyadh, and gynecology departments at two hospitals, namely Medicare Cardiac and General Hospital and Fatima Bai Hospital in Karachi, Pakistan. The departments were chosen in view of their high readiness to accept and implement connected health technology in their provision of care. For instance, the bariatric surgery department is a center for excellence and has been considered a model for initiating this program in terms of staff and resources.

The Home Healthcare Department at KSUMC, Riyadh provides long-term health and medical care and treatment at home to the people aged 60 years or older and are terminally ill or the oldest old (80+ years) population with or without any serious health conditions. Generally, one physician and one or two nurses perform a group visit to each patient once a month to perform clinical examination and follow-up, and the procedure includes paper-based monitoring and recording system. There are around 200 patients being provided home-based health care by two physicians and three nurses in the department. The idea of incorporating a connected health intervention into clinical care in the Department of Home Healthcare, KSUMC is to make the home-based long-term care more efficient and effective. A target of recruiting 40 patients out of 200 home healthcare patients has been set for the RAHAH intervention and, so far, 22 patients have been enrolled.

## Intervention providers

RAHAH involves three different groups of experts: (a) physicians or nurses; (b) execution team; and (c) operations/developers. First, a group of physicians and nurses provide continuous monitoring of the patients, maintain follow-up sessions and regularly communicate with them. The execution team, on the other hand, continuously monitors the intervention, trains, provides support to the physicians and nurses, tests new features, and collects all the feedbacks. This team consists of a computer science engineer, a patient coordinator with proficiency in health information management and technology, a health education specialist and a technical supervisor. Lastly, the developers are the ones who develop apps, add new features and fix issues. There are 4 portal developers, 2 iOS App developers, 2 Android App developers, 2 Telemedicine developers and 2 webinar developers.

## Materials being used in the intervention (the what)

RAHAH incorporates the use of various digital health devices and technologies by both the patients who are being monitored and the intervention providers. A summary description of the devices is outlined in Table 1.

**Table 1:** Summary of RAHAH devices and technologies

Devices	Description	Use
1. Patient Portal and Physician Portal	It is a secure website for both the patient’s and healthcare providers. The patient portal dashboard consists of a Profile (which contains the patients basic and emergency information), Observations, Health Records, Lab and Reports, Educational feature, Telemedicine, Webinar, Messages and Chat. The doctor portal dashboard consists of the patient profile, observation (reading from patient devices), patient health record, doctor’s notes (for typing notes for the patient and viewing notes of other doctors), lab reports, heart sound and lung sound, patients vital signs statistical analysis, e-Response Center, Messaging, Appointment schedules, Telemedicine and Webinar.	It allows both the patient and healthcare provider to create and monitor personal health records, view readings from the devices, and communicate with each other.
2. Remote Devices (glucometer, blood pressure monitor, pulse oximeter, thermometer and body analysis scale). For pregnancy monitoring, fetal beat and kick track are used.	Based on the clinical condition of the patients, these devices are recommended by physicians and consultants for regular remote monitoring of the patients. Patients use the devices and data generated from them will be transferred via Bluetooth to their mobile Apps and into the RAHAH portal.	These devices are used to remotely monitor health/disease conditions, and vital signs of the patients.
3. Mobile Applications (mApps)	The RAH@H mApps are vendor agnostic (iOS/android) in nature and hence work on any brand of smart phone. Moreover, RAH@H has separate mApps for patients and healthcare providers, thus there are four different types of mobile apps present in RAHAH.	RAHAH app is used to access the individual patient portal and transfer readings, which are crucial for monitoring.
4. Patient Health Records (PHR)	An electronic health record in which the profiles for each patient are created and their medical information are recorded (e.g., lab tests, X-rays).	PHR centralizes all (past and present) health-related information of the patient. (e.g., medications, allergies, health conditions, surgical procedures, family history etc.) of the patient.
5. Telemedicine	Both the patients and providers can arrange an audio-visual teleconsultation either through appointments or directly. Data generated from remote monitoring medical devices which are recorded in the PHR helps to better understand the patient’s condition and makes the consultation more effective. Moreover, past online consultations can be reviewed, health reports can be made visible and anatomical pictures can be shown for further clarification.	Access to remote specialist care, consultation and follow-up can be arranged immediately.
6. E- Response Center (ERC)	The ERC receives alerts and notifications for any alarming (danger) signs so that immediate actions can be taken. It is controlled by an appropriately trained staff in respective departments.	Detection of any alarming signs can be immediately discussed with the patient/provider though teleconsultation, chat or messaging.
7. Webinars	The patients receive the links for webinars that are conducted by specialists, health educators, doctors, nutritionists and nurses in the respective area. Patients can join live as well as view archived broadcasts. The webinar presenter can invite a co-presenter, share PowerPoints, videos and screen. Surveys can be conducted for patients to participate.	Patients are given educational information and can ask experts audio, video or text based queries regarding disease, symptoms, medication use, prevention, wellness, and nutrition.
8. User Generated Content	The patients and health care providers can read/watch/share articles, blogs and videos according to their specific health condition for latest updates and news.	Patients get informed and educated to better understand the disease and symptoms, and self-care approaches.
9. Encyclopedia	Patients can access a wide range of information about their medical condition such as diagnostic tests, symptoms, injuries, surgery and wellness through interactive tutorials and illustrations via health encyclopedia. Patients can also take part in short quizzes after each tutorial and can download all the information in pdf format.	Patients can get access to relevant, reliable, and easy to understand health information. It is also available in Arabic.
10. Online peer groups	The patients can interact and share learning materials with other patients.	Patients can access the information and support in the group.

## Intervention procedures (the how)

Especially, in the Home Healthcare Department, KSUMC, the intervention would follow the following three steps:

### Step 1. Patient enrolment

In the Home Healthcare Department, KSUMC, remote devices are used to monitor terminally ill or geriatric patients, especially those who are also clinically confirmed of being hypertensive or diabetic or both (clinical eligibility). The patients who meet this clinical eligibility criteria are then further checked if other non-clinical eligibility criteria are met for enrollment in the intervention.

The non-clinical eligibility criteria are as follows: (a) the patient/caregiver should have basic English language skills, (b) the patient/caregiver should have computer and web browsing skills or, is familiar with smart phones and mobile apps, (c) the patient/caregiver should confirm the availability of a computer or smartphone and (d) the patient/caregiver confirms access to 24/7 internet connection.

The patient/visitor who meets both the clinical and non-clinical eligibility criteria is enrolled in the intervention and is then explained about the intervention and are requested for a voluntary written informed consent to confirm their participation. Next, each enrollee is provided with bar-code recorded medical devices based on their clinical requirements, and a system-generated user-name and password that would enable them to log into their personal portal.

### Step 2. Training to the patients/visitors

The physicians or nurses in the Home Healthcare Department, KSUMC, provide instructions to the enrollees (who are more often the families and caregivers in the home health care department since patients are usually in no condition to participate themselves) and ask them to demonstrate how to use remote medical devices, patient portal, telemedicine, and mobile apps and ask to demonstrate using the devices. Physicians and nurses from the Home Healthcare Department have received a training about how to provide instructions and demonstrations and describe how to appropriately use connected health devices and technologies.

### Step 3. Remote patient monitoring

In the Home Healthcare Department, the enrollees are monitored for a trial period for several days to make sure whether they have understood well and can independently use patient portal, devices and telemedicine. They are also provided with an access to additional manuals and videos for more information on how to use the devices. The monitored patients use the remote medical devices (to measure the blood pressure and blood sugar), and transfer the information two times a day. In case, if anything abnormal is detected, the physicians and the patients will be able to track the data and communicate immediately.

Adherence of the patient to the treatment and management plan as per the physician recommendation is monitored on the RAHAH staff portal by the physicians, nurses and the execution team. Patients who fail to take their readings for more than two days are sent reminders through RAHAH messaging feature or phone calls by the RAHAH execution team.

Tele-consultation sessions are scheduled biweekly and are carried out by two physicians in both Arabic and English language to remotely provide consultation and follow-up regarding the patient’s health condition and view the progress of specific conditions such as healing of bed sores and medicines are prescribed accordingly. Webinar sessions are organized once a week, and the patients/visitors also have an access to additional information through encyclopaedia that together provide information on topics such as nutritional related guidelines, foot care for diabetic patients, care for hypertension, hypoglycaemia and prevention of falls in the elderly.

## Intervention modifications and tailoring

RAHAH is still evolving and has not undergone any significant intervention modification process. To document the change process, the execution team organizes regular meetings and seminars and maintains technical and communication logs. Feedback surveys to understand the enrollees’ satisfaction and their adherence to the intervention are being planned to generate some evidence on the quality of remote patient monitoring process. Since the intervention is still in a development phase, fidelity has not been assessed yet. Moreover, RAHAH has been conceptualized to be tailored to provide personalized health care by continuous monitoring of patients, supporting treatment adherence and providing individualized counselling and information to the patients who need them the most.

## Discussion

RAHAH is a connected health intervention with a primary aim to remotely monitor the patients needing long-term medical care in Saudi Arabia and Pakistan. RAHAH can be considered as a very comprehensive model since it has introduced some new and important features for patient monitoring and education, including user generated content, webinars and encyclopedia. In addition, RAHAH is constantly evolving its features, which is believed to make it easier for healthcare providers to comprehensively engage, monitor, treat and educate the patients and empower them towards behavior change and self-care approaches. RAHAH can make an important contribution to integrate remote medical device-based patient monitoring in the mainstream health care, and reduce the total burden of health care costs in Saudi Arabia and Pakistan.

We also recognize the fact that RAHAH being a multi-country connected health intervention may face serious financial, logistical, technical, and human resource challenges in terms of its successful implementation. However, no evidence on the challenges of a connected health intervention in the context of Saudi Arabia and Pakistan, has been documented so far. Based on inadequate available evidence, we have identified the challenges and provided below some of our reflections on how we should plan to tackle those challenges:

First and foremost, we anticipate that the simultaneous implementation of RAHAH in two contextually different countries would provide some important insights into what factors would influence the implementation of connected health interventions [27]. For instance, there could be huge dissimilarity in terms of structural factors, such as access to resources, between these two countries, which could further influence the level of individual patients’ motivation to manage their health and their willingness to be an active participant in their healthcare [28]. It is also important to emphasize that RAHAH should consider the socio-structural and individual factors during the implementation and evaluation of RAHAH in the future.

Secondly, from an intervention provider point-of-view, RAHAH clearly warrants the need for both, clinical and non-clinical staff to be motivated and equipped with necessary e-health skills to deal with the regular as well as unforeseen situations during the intervention implementation. For instance, Boonstra and Broekhuis (2010) have noted that, despite the positive effects of e-health usage in medical practices, the adoption rate of such systems is still low and could face resistance from physicians [29]. Thus, successful implementation of RAHAH may also include strong and continuous political will on the part of the leadership and provision of adequate financial resources required to support long-term successful program implementation.

Thirdly, we recognize the fact that a comprehensive and complex connected health intervention can only be possible through international collaboration and partnership. Therefore, we have sought for international collaborations for developing and monitoring the intervention tools, training, competency development, and expertise resourcing. We are also aware of the fact that managing these collaborations requires well-planned strategies to develop functional communication channels at all stages of collaboration [26]. The long-term challenge would be to develop strategies for scaling up of the intervention and integrating it into the mainstream healthcare. For this, one of the proven strategies is to inform the implementation and evaluation of the intervention by evidence generated by transdisciplinary research works using not just quantitative but also qualitative and mixed method research types [30]. Recognizing this fact, Prince Naif Health Research Centre has been formed at KSUMC for in-house coordination of all the research works and international research collaboration. Further strategies and opportunities will be sought to scale up this connected health intervention.

Next, it should clearly be noted that RAHAH has been limited in enrolling the patients with English language skills and those who have a good digital health literacy. We recognize that an important section of the population that might particularly benefit from remote patient monitoring could have been left out because of our strict non-clinical recruitment criteria. It has been also noted by the work of Dashti et al. (2017) in Iran that e-health literacy tends to be poorer among women and those who are illiterate and have a low income [31]. Therefore, we are planning to conduct a study on examining healthcare workers’ intentions to provide remote monitoring service and the e-literacy level of the people which would further help to determine which section of population should be targeted by RAHAH. This information is believed to be crucial to identify the strategies that should be considered while scaling up the intervention to a wider population, especially in Saudi Arabia [32].

Lastly, evidence on how to implement new theoretically informed interventions into complex healthcare environments is often poorly reported and indexed, reducing its potential to inform initiatives to improve healthcare services [33]. Although RAHAH has been informed by several theoretical frameworks for behaviour change, empowerment and adherence, the manuscript has not referenced any frameworks. We believe, the theoretical aspects of the intervention design and implementation should be better illustrated by future research works related to RAHAH.

## Conclusion

In this paper, we discussed the components of RAHAH and how it has been implemented in Saudi Arabia and Pakistan in order to provide remote medical device-based monitoring of the patients who need long-term medical care. In addition, we provide insights about the challenges of implementation and discussed some strategies to overcome them. We believe, RAHAH would turn out to be a very important connected heath intervention to better manage a growing chronic care population in Saudi Arabia and Pakistan, and the lessons learnt might be applicable elsewhere.

## Abbreviations

ERC: e Response Center
HIV: Human Immunodeficiency Virus
KSUMC: King Saud University Medical City
mApps: Mobile Applications
PHR: Personal Health Record
RAHAH: Remotely Accessible Health Care at Home
TB: Tuberculosis
TIDieR: Template for Intervention Description and Replication

## References

[1] Altuwaijri MM (2008). Electronic-health in Saudi Arabia. Just around the corner?. Saudi Med J..

[2] Terada T, Nakamura K, Seino K, Kizuki M, Inase N (2018). Cost of shifting from healthcare to long-term care in later life across major diseases: analysis of end-of-life care during the last 24 months of life. JRM..

[3] Rosman M, Rachminov O, Segal O, Segal G (2015). Prolonged patients’ in-hospital waiting period after discharge eligibility is associated with increased risk of infection, morbidity and mortality: a retrospective cohort analysis. BMC Health Serv Res.

[4] Harkanen M, Kervinen M, Ahonen J, Voutilainen A, Turunen H, Vehvilainen-Julkunen K (2015). Patient-specific risk factors of adverse drug events in adult inpatients - evidence detected using the global trigger tool method. J Clin Nurs.

[5] Vegesna A, Tran M, Angelaccio M, Arcona S (2017). Remote patient monitoring via non-invasive digital technologies: a systematic review. Telemed J E Health.

[6] Singh K, Drouin K, Newmark LP, Rozenblum R, Lee J, Landman A, Pabo E, Klinger EV, Bates DW (2016). Developing a framework for evaluating the patient engagement, quality, and safety of Mobile health applications. Issue brief (Commonw Fund).

[7] Barton AJ (2012). The regulation of mobile health applications. BMC Med.

[8] Caulfield BM, Donnelly SC (2013). What is connected health and why will it change your practice?. QJM-Int J Med.

[9] Argent R, Daly A, Caulfield B (2018). Patient involvement with home-based exercise programs: can connected health interventions influence adherence?. JMIR Mhealth Uhealth.

[10] Dillingham R, Ingersoll K, Flickinger TE, Waldman AL, Grabowski M, Laurence C (2018). PositiveLinks: a Mobile health intervention for retention in HIV care and clinical outcomes with 12-month follow-up. AIDS Patient Care STDs.

[11] Ybarra ML, Eaton WW (2005). Internet-based mental health interventions. Ment Health Serv Res.

[12] Novillo-Ortiz D, Dumit EM, D'Agostino M, Becerra-Posada F, Kelley ET, Torrent-Sellens J (2018). Digital health in the Americas: advances and challenges in connected health. BMJ Innov.

[13] Hoffmann TC, Glasziou PP, Boutron I, Milne R, Perera R, Moher D (2014). Better reporting of interventions: template for intervention description and replication (TIDieR) checklist and guide. BMJ (Clin Res Ed).

[14] Almutairi KM, Moussa M (2014). Systematic review of quality of care in Saudi Arabia. A forecast of a high quality health care. Saudi Med J.

[15] Almalki M, Fitzgerald G, Clark M (2011). Health care system in Saudi Arabia: an overview. East Mediterr Health J.

[16] Sabra SM, Abdel-Fattah MM (2012). Epidemiological and microbiological profile of nosocomial infection in Taif hospitals, KSA (2010-2011). WJMS..

[17] Sebai ZA, Milaat WA, Al-Zulaibani AA (2001). Health care services in Saudi Arabia: past, present and future. J Fam Community Med.

[18] Alharbi MF (2018). An analysis of the Saudi health-care system's readiness to change in the context of the Saudi National Health-care Plan in vision 2030. Int J Health Sci.

[19] Health Outreach.King Faisal Specialist Hospital & Research Centre. 2018. https://www.kfshrc.edu.sa/en/home/aboutus/healthoutreach Accessed on 8 Aug 2018.

[20] World Health Organization: Noncommunicable diseases Key Facts. https://www.who.int/news-room/fact-sheets/detail/noncommunicable-diseases (2018). Accessed 29 Dec 2018.

[21] World Health Organization: Pakistan WHO statistical profile. http://www.who.int/gho/countries/pak.pdf?ua=1 (2015). Accessed 15 July 2018.

[22] Majrooh MA, Hasnain S, Akram J, Siddiqui A, Shah F, Memon ZA (2013). Accessibility of antenatal services at primary healthcare facilities in Punjab, Pakistan. J Pak Med Assoc.

[23] Nishtar S, Boerma T, Amjad S, Alam AY, Khalid F, Haq I (2013). Pakistan's health system: performance and prospects after the 18th constitutional amendment. Lancet..

[24] National Health Vision Pakistan. 2016–2025. http://www.nationalplanningcycles.org/sites/default/files/planning_cycle_repository/pakistan/national_health_vision_2016-25_30-08-2016.pdf. Accessed 17 July 2018.

[25] United Nations: Sustain Dev Goals. https://www.un.org/sustainabledevelopment/infrastructure-industrialization/ Accessed 18 July 2018.

[26] Varshney D, Atkins S, Das A, Diwan V (2016). Understanding collaboration in a multi-national research capacity-building partnership: a qualitative study. Health Res Policy Syst.

[27] Elachola H, Memish ZA (2016). Oil prices, climate change—health challenges in Saudi Arabia. Lancet..

[28] Hibbard JH, Greene J (2013). What the evidence shows about patient activation: better health outcomes and care experiences; fewer data on costs. Health Aff (Project Hope).

[29] Boonstra A, Broekhuis M (2010). Barriers to the acceptance of electronic medical records by physicians from systematic review to taxonomy and interventions. BMC Health Serv Res.

[30] Maglaveras N, Kilintzis V, Koutkias V, Chouvarda I (2016). Integrated care and connected health approaches leveraging personalised health through big data analytics. Stud Health Technol Inform.

[31] Dashti S, Peyman N, Tajfard M, Esmaeeli H (2017). E-health literacy of medical and health sciences university students in Mashhad, Iran in 2016: a pilot study. Electron Physician.

[32] Zakaria N, AlFakhry O, Matbuli A, Alzahrani A, Arab NSS, Madani A (2018). Development of Saudi e-health literacy scale for chronic diseases in Saudi Arabia: using integrated health literacy dimensions. Int J Qual Health Care.

[33] Ross J, Stevenson F, Dack C, Pal K, May C, Michie S (2018). Developing an implementation strategy for a digital health intervention: an example in routine healthcare. BMC Health Serv Res.

